# Nurses' Trials—Responsibility

**Published:** 1887-03-19

**Authors:** 


### WORDS OF CONSOLATION.

[Communications for this column should be addressed, "Chaplain," care of the Editor of THE HOSPITAL, who will gratefully welcome any suggestions or inquiries from matrons, nurses, and the staff generally.]

### XXV.—NURSES' TRIALS—RESPONSIBILITY.

"IF you can find a place between the throne of God and the dust to which man's body crumbles, where the focal responsibilities of law do not weigh upon him, I will find a vacuum in nature. They press upon him from God out of eternity, and from earth out of nature, and from every department of life, as constant and all-surrounding as the pressure of air." (Beecher.)

"Some people may shirk responsibility, none can really escape it. The pressure varies according to the character of the bearer. Timid and fearful natures, who would shift responsibility on to other shoulders than their own, forget that they are responsible for the transfer they elect to make. We may be tempted to shield ourselves by the thought of leaving all we dare to others. But in leaving to others duties which conscience once proclaimed to be our own, we probably have incurred much greater responsibility, than if we boldly made the attempt ourselves to do them, and yet failed. Responsibility is by no means so easily transferable as we conceive." (Author of *Amy Herbert.*)

Those whom we addressed last week as feeling responsibility so deeply as to find it a trial, are not the most likely to attempt such a transfer. The well-meaning, but indolent, or thoughtless, less serious minds are the ones so apt to yield to this side of temptation; especially in little duties. They perhaps hold themselves responsible up to a certain point—say, for what they must do, or for what forms the palpable portions of work for which they were engaged, or for just what no one else in the same office is likely to do but themselves. But as soon as they have to face less direct obligations their moral sense seems blunted. In a word, their sense of responsibility does not extend beyond stipulated bounden duty and service. Now such a hard and limited view as this may be pardonable amongst the less educated, but it is hardly admissible in those who have devoted themselves to such sacred work as tending the sick

Probably, in every large establishment the machinery of work runs more smoothly where a majority consider themselves responsible for helping whenever they can, in matters which do not strictly concern their obligations. "Bear ye one another's burdens, and so fulfil the law of Christ" (Gal. vi, 2). Directly this divine precept is lost sight of by persons much associated in work, friction is certain to arise. To make one's responsibility extend to others burdens is not really to increase the weight of our own. Just the reverse is the truth. By striving to increase a feeling of mutual interdependence we find our own stipulated obligations are safer and surer of fulfilment, because others will be watching and willing to lend help in times of need. Of course there are persons to be found engaged in nearly every good work who have too great a sense of their own importance, and consequently give offence by intruding or trenching upon duties not strictly their own. They have not learnt to keep their place. We are more concerned now with evasions than intrusions. We say, therefore, to all paid nurses, especially to those who have very precise notions of the exact work they were engaged to do: By all means look upon this as your primary obligation; but do not draw the line of your moral responsibility at this point. Draw it higher up for Christ's sake. Try and feel that your present training is an education for some far greater responsibility in the kingdom to come, wherein all obligation will give way for ever to the reign of love.

